# Association of Kidney Disease, Potassium, and Cardiovascular Risk Factor Prevalence with Coronary Arteriosclerotic Burden, by Sex

**DOI:** 10.3390/jpm11080722

**Published:** 2021-07-27

**Authors:** Patricio Maragaño Lizama, Diana L. Ríos, Isaac Subirana Cachinero, Andrea Toloba Lopez-Egea, Anna Camps, Oward Belzares, Claudio Pacheco, Cristina Cerro, Sergio Wehinger, Eduardo Fuentes, Jaume Marrugat, Iván Palomo

**Affiliations:** 1 Unidad de Hemodinamia, Hospital Regional de Talca, Talca 3460000, Chile; pmaraliz@gmail.com (P.M.L.); owardbelzarezmd@gmail.com (O.B.); cmpachecoc@outlook.cl (C.P.); ccerroc@hospitaldetalca.cl (C.C.); 2 Thrombosis Research Center, Department of Clinical Biochemistry and Immunohematology, Faculty of Health Sciences, Medical Technology School, Universidad de Talca, Talca 3460000, Chile; diana.rios@utalca.cl; 3 REGICOR Research Group, IMIM (Hospital del Mar Medical Research Institute), 08025 Barcelona, Spain; isubirana@imim.es (I.S.C.); atoloba@imim.es (A.T.L.-E.); acamps@imim.es (A.C.); jaume@imim.es (J.M.); 4 CIBERESP de Investigación en Epidemiología y Salud Pública, 28001 Madrid, Spain; 5 CIBERCV de Investigación en Enfermedades Cardiovasculares, 28001 Madrid, Spain

**Keywords:** cardiovascular diseases, cardiovascular risk factors, coronary artery disease, ST-elevation myocardial infarction, percutaneous coronary intervention, atherosclerotic lesion

## Abstract

The present study aimed to determine the relationship between the prevalence of cardiovascular risk factors and the number and severity of coronary artery atherosclerotic lesions obtained by coronary angiography. We reviewed and analyzed 1642 records from consecutive patients at the Catheter Laboratory of Talca Regional Hospital in Chile between March 2018 and May 2019. Patients were stratified according to the presence and severity of atherosclerotic lesions: 632 (38.5%) had no lesions or <30% stenosis and 1010 (61.5%) had at least one coronary atherosclerotic lesion with ≥30% stenosis (CALS-30). CALS-30 was more frequent in males, smokers, and patients with diabetes and/or hypertension (all *p*-values < 0.02). Serum potassium, glycaemia, creatinine and glomerular filtration rates were also associated with CALS-30 (all *p*-values < 0.01) in males. The age and the proportion of males with CALS-30 increased with the number of risk factors (*p*-values for trends < 0.001). Our results showed a stronger association between the accumulation of risk factors and CALS-30 in women than in men. Serum potassium levels were inversely associated with CALS-30 in men but not in women.

## 1. Introduction

Cardiovascular diseases (CVDs) currently account for nearly half of all noncommunicable diseases worldwide [1] and constitute the leading cause of death, taking an estimated 17.9 million lives each year [2]. Coronary artery disease (CAD) is the main CVDs-related cause (43.8%) of death, followed by cerebrovascular accidents (16.8%), heart failure (9%), high blood pressure (9.4%), arterial diseases (3.1%), and other vascular diseases (17.9%) [3].

CAD is often caused by a buildup of plaque in the subendothelial space of coronary arteries, which limits or blocks the blood supply to the myocardial muscle [4,5]. For decades, atherosclerotic lesions in the coronary arteries have been related to the burden of cardiovascular risk factors (CVRF). Some are unmodifiable, such as CAD family history, age, sex, ethnicity, or socioeconomic status, but most are modifiable. These include high total and low-density lipoprotein (LDL) cholesterol, low levels of high-density lipoprotein (HDL) cholesterol, smoking, hypertension, diabetes, impaired glucose tolerance, obesity, and sedentarism, which contribute to CAD in varying degrees [6,7,8,9,10,11,12].

In Chile, the 10-year CAD incidence is 2.7% in males and 1.1% in females, and this percentage is much lower than in the United States and Northern Europe. The highest CAD risk in Chile has been observed in diabetic male smokers and the lowest in nondiabetic females [13]. The most prevalent CVRF in adults in Chile includes a sedentary lifestyle (90.8%), cigarette smoking (42.0%), low HDL-cholesterol (39.3%), hypercholesterolemia (35.4%), hypertension (33.7%), and obesity (23.2%) [14].

Worldwide, CAD management has improved in recent decades as a response to the increasingly high incidence and case-fatality rates observed in the second half of the 20th century [15]. Coronary angiography and percutaneous coronary intervention (PCI) have been extensively used to diagnose and treat CAD for the last 30 years [16].

Although the relationship between CVRF and CAD has been extensively confirmed, its association with the number and severity of coronary atherosclerotic lesions in vivo, and particularly with subclinical lesions with <50% stenosis without provoking myocardial ischemia in a stress test still requires further investigation.

The purpose of this study was to evaluate the relationship between the prevalence of CVRF and the presence and severity of coronary atherosclerotic lesions as determined by coronary angiography.

## 2. Materials and Methods

### 2.1. Study Design

A cross-sectional study was performed to determine the association between CVRF prevalence and coronary atherosclerosis burden in 1642 records from consecutive patients admitted at the catheter laboratory of the Talca Regional Hospital between March 2018 and May 2019.

### 2.2. Study Population

All consecutive patients referred from public and private hospitals in the Maule Region (900,000 inhabitants) to the Talca Regional Hospital were considered for inclusion in the study group. Patients with ST-elevation myocardial infarction (STEMI), non-STEMI, unstable angina, chronic angina, and preoperative valve surgery were selected for analysis from the regional database anonymized for research use. Standard CAD definitions were used [17]. After excluding 193 patients who had a history of myocardial infarction (>30 previous days), 1642 patients who had consented to participate in the study were retained for analysis.

### 2.3. Measurements

Demographic variables, history of CVRF (i.e., hypertension, diabetes mellitus, smoking at least one cigarette/day currently or in the last 12 months, dyslipidemia, body mass index [BMI], chronic kidney disease [CKD]), and previous CVDs procedures (e.g., angioplasty, surgical myocardial revascularization) were recorded. The results from routine blood tests (glycaemia, creatinine, potassium, and blood urea nitrogen [BUN]) were also collected from the anonymized database. The glomerular filtration rate (GFR) was calculated by the Chronic Kidney Disease Epidemiology Collaboration method (CKD-EPI) [18].

Three trained interventional cardiologists assessed the number and severity of lesions in four main coronary arteries according to common criteria. The number of lesions and their observed degree of vessel stenosis were reported for the left main coronary artery (LM), anterior descending artery (LAD), circumflex artery (CX), and right coronary artery (RCA). The total number of lesions and a cutoff-point at 30% stenosis were used to classify participants into two groups: at least one coronary atherosclerotic lesion with ≥30% stenosis (CALS-30) or no lesions or <30% stenosis (non-CALS-30) were calculated. The catheter laboratory used Allura Xper FD Phillips equipment and the laboratory team did not change during the study period.

### 2.4. Statistical Analysis

Categorical variables were summarized using counts and percentages. Continuous variables were summarized by the mean and standard deviation (SD), or the median and corresponding interquartile range. Bivariate tables are presented to test the association of values for the continuous variables with CALS-30 status and with risk factor prevalence. Associations were tested with Chi-Square, Student t-test, or equivalent nonparametric tests as appropriate. Logistic regression models were fitted to determine the age- and sex-adjusted effect (odds ratio [OR]) with a 95% confidence interval (95% CI) of CALS-30 for the individual risk factors and as a score of the accumulated number of risk factors (smoking, CKD, diabetes, and hypertension). For ordinal variables (e.g., the risk factors score) the association with categorical and continuous variables was tested with Chi-Square for trends and linear regression, respectively. The *p*-value threshold for statistical significance was 0.05.

### 2.5. Ethical Issues

The study protocol was approved by the Scientific Ethics Committee from the University of Talca (N°2016-019-IP). The database was anonymized for research use.

## 3. Results

The demographic and CVRF characteristics of participants are shown in Table 1 by CALS-30 status. Of the 1642 records studied (556 women [33.1%] and 1086 [66.1%] men), 1010 (61.5%) had at least one CALS-30, and 632 (38.5%) had none. There was no difference in age between the groups with or without CALS-30. However, when stratified by sex, the CALS-30 group was significantly older. Men over 55 and women over 65 years showed significantly higher CALS-30 than younger patients (Supplementary Tables S1 and S2). Women were less likely to have CALS-30 than men (Table 1).

Smoking, hypertension, diabetes, history of the previous PCI, and CKD or GFR <60 mL/min were significantly associated with CALS-30. High BMI and obesity were inversely associated with CALS-30 (Table 1). By sex, women showed a greater association between the percentage of significant lesions and hypertension compared to men. Glycaemia, creatinine, GFR < 60 mL/min, and potassium were significantly associated with CALS-30, both overall and when stratified by sex. In contrast, differences between CALS-30 groups in GFR < 60 mL/min, glycaemia, and creatinine remained significant in women, as did potassium and glycaemia differences in men (Supplementary Tables S1 and S2).

Table 2 shows the indications for coronary angiography according to patient CALS-30 status. Emergency and urgently programmed angiographies were more frequent in CALS-30 patients, who were also more frequently diagnosed with STEMI, non-STEMI, and unstable angina than their non-CALS-30 counterparts.

In the multivariate logistic regression, age, male sex, smoking, diabetes, and CKD were found to increase the risk of CALS-30. Hypertension showed no significant differences in the overall analysis, but when stratified by sex (Supplementary Tables S3 and S4), this was associated with a higher risk of lesions in females. Women also had an increased risk of lesions associated with smoking, diabetes, and renal failure; in men, an increased risk was associated with age, smoking, and diabetes.

Given the significant association observed between CALS-30 and serum potassium levels (Table 1), a multivariate analysis adjusted for potassium was performed. The five identified risk factors (age, smoking, diabetes, hypertension, and renal failure) retained significance, and higher serum potassium levels were independently associated with a lower risk of CALS-30 (Table 3). When stratified by sex, this association persisted only in men (Supplementary Tables S5–S8).

Next, the relationship between the number of accumulated CVRF and age, sex, BMI > 30, previous PCI, and laboratory test results were evaluated. CALS-30, age, and the proportion of women significantly increased with the number of accumulated CVRF (Table 4).

**Figure 1:**
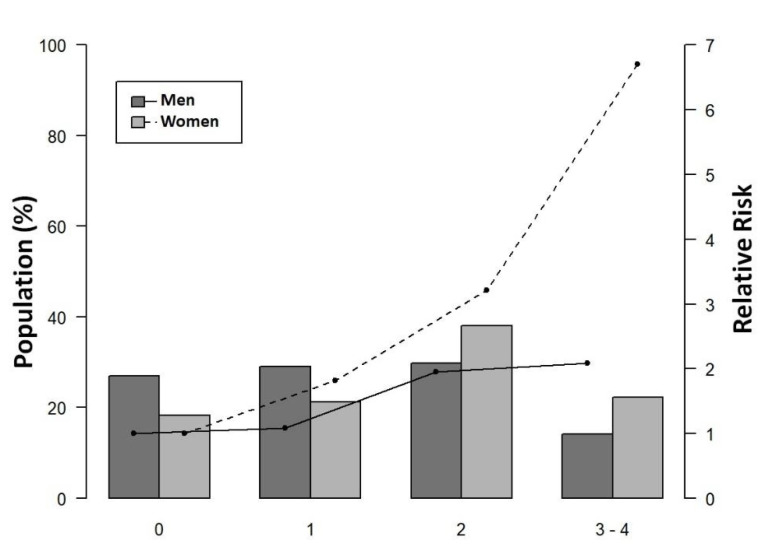
Population distribution, by sex, of the accumulated number of classical cardiovascular risk factors and risk of the presence of atherosclerotic coronary lesions with **≥**30% stenosis, based on coronary angiography.

The age- and sex-adjusted risk of CALS-30 significantly increased with the number of accumulated CVRF in females (Table 5). The impact of the number of accumulated risk factors on the crude risk of CALS-30 was more influential in females than in males, notably at three or four risk factors (Figure 1).

## 4. Discussion

Although coronary stenosis ≥50% or even ≥70% is generally accepted as significant based on its potential to cause provocable myocardial ischemia [19,20], the 30% threshold was selected to include a wide range of asymptomatic small lesions producing stenoses ranging from 30% to 70%, which typically have been excluded in previous studies for a purely clinical reason: these lesions seldom generate provocable myocardial ischaemia [20]. However, to better show the effect of risk factors at a population scale, we chose to identify people with atherosclerotic lesions rather than with only severe lesions or symptomatic CAD disease. The number of patients in each subgroup of lesion size changed only slightly from 1010 with a CALS-30 cutoff-point of 30% stenosis to 969 with lesions ≥50% stenosis and a bit lower for lesions ≥70% stenosis (Supplementary Tables S9–S14). The originality of our work relies on the fact that we did not exclusively analyze the relationship between the cardiovascular risk factor burden with symptomatic coronary artery disease (i.e., coronary occlusions ≥70%) but also that with asymptomatic coronary atherosclerotic lesions (i.e., lesions with ≥30% to 69% occlusion), which are commonly neglected and rarely studied. We compared the risk factor burden of all these patients with that of subjects with no or very small lesions (i.e., <30% occlusion). This was done by invasive methods and by taking advantage of a large number of patients with angiographies with lesions with <70% occlusion available at the catheter laboratory in Talca.

Intriguingly, no significant difference in the mean age between the two groups was found, but the number of males older than 55 years and females older than 65 years was significantly higher in the group with lesions with ≥30% stenosis. The CALS-30 group had a greater proportion of males, as expected due to the known difference in CAD incidence/prevalence between males and females. Women are known to present less obstructive epicardial stenosis but more diffuse atherosclerosis and microvascular dysfunction than men [21] and are more likely to have less severe CAD than men of a similar age [22,23].

Our results also suggest sex-based differences in the effect of the CVRF profile. Although men had CALS-30 more often, the prevalence of risk factors was higher in women. In addition, the accumulated number of risk factors increased the risk of CALS-30 more intensely in women than in men. It has been proposed that women have significant differences in artery dimension and atherosclerotic plaque composition and development compared to men, so the impact of cardiovascular risk factors might also be different [24]. The CVRF profile is usually less favorable in men in terms of cardiovascular events [25], but the presence of major CVRF has also been found to result in worse outcomes in women [26]. Indeed, smoking, diabetes, and low HDL cholesterol are known to be significantly more hazardous for women than for men in CAD development [25,27,28]. The novelty of our results is the association observed with CALS-30, which is likely an intermediate early marker for future CAD events. Nevertheless, the pathophysiology of these differences remains unclear.

The association between diabetes and CVDs has been widely documented, and in CVDs the risk of diabetes is considered equivalent to that of CAD patients [29]. Our results showing diabetes as a strong predictor of CALS-30 in both sexes agree with Xu et al., who reported that 55.2% of diabetic patients were found to have coronary lesions and 16.4% had significant stenosis detected by coronary computerized tomographic angiography [27]. Our results showed that 48.6% of patients with CALS-30 had diabetes or glycaemia >126 mg/dL.

We also observed that ex-smokers <1 year had a significantly higher risk for injuries of ≥50% and ≥70% stenosis but not for CALS-30 (Supplementary Tables S9 and S10). One possible explanation is the known association of tobacco use with increased platelet aggregation and plaque instability [30]. Another possibility is that patients had experienced coronary symptoms that led them to stop smoking within the year before the study period.

We also observed that serum creatinine levels were associated with CALS-30. Our findings on CALS-30 lesions parallel those of Bagheri et al. on the association of serum creatinine with the prevalence and intensity of CAD [31]. In women, lower GFR was independently associated with CALS-30.

Impaired kidney function favors the progress of atherosclerosis, notably increasing the risk of cardiovascular events and worsening prognosis in patients with CVDs [32]. However, in our case, blood urea nitrogen (BUN) levels were not associated with significant lesions. This is not entirely unexpected, as BUN levels change much faster than creatinine and GFR, making BUN a less reliable early marker for assessing chronic kidney damage than creatinine and, in particular, GFR. However, elevated BUN levels have been associated with increased long-term mortality from CVDs in patients with PCI, independently of traditional CVRF and GFR [33]. In that report, however, the mean BUN level was >25 mg/dL, while in our study the average value was 16.4 mg/dL regardless of CALS-30 status.

A very interesting and unexpected finding in the present study was the independent association of decreased serum potassium levels with CALS-30. Potassium levels have a narrow range of normality, and alterations are usually associated with kidney disease.

The described serum potassium levels show a U-shaped association with cardiovascular death with harmful high and low levels [34]. An abnormally high serum potassium concentration (i.e., ≥5.0 mEq/L) has been associated with cardiovascular mortality, especially in diuretic users [35], and it has been suggested that early detection and treatment of hyperkalemia may reduce the risk of sudden cardiac death in patients with CKD [36]. In contrast, our results linked decreased levels of potassium to CALS-30 in patients within normal kalemia ranges. In this respect, a decreased level of potassium has been previously related to CVD. Low-normal serum potassium has been associated with a significantly increased risk of cardiovascular mortality in an elderly community [37]. Low levels of potassium are linked to vascular calcification, contributing to atherosclerosis in a cAMP response element-binding protein (CREB) and in an autophagy-dependent manner, as reported in mouse studies [38]. Additionally, a high sodium/potassium excretion ratio has been proposed as an indicator of an increased risk of CVD, and this is associated with high-in-sodium/poor-in-potassium diets [39]. We found a significant independent association between lower serum potassium levels and the presence of coronary atherosclerotic stenosis lesions in non-hyperkalemic conditions, suggesting a potential indicator of increased CALS-30 risk. It is important to stress that this association was within a normokalemic range, so other factors could have influenced this, making cardiovascular conditions more sensitive to changes in potassium homeostasis. Low potassium levels have been related to several disorders, including cardiac arrhythmias, sudden cardiac death, cellular free radical formation, vascular smooth muscle cell proliferation, arterial thrombosis, and augmented vascular adherence by macrophages, leading to vascular lesions [40]. However, the pathophysiology behind these associations and their possible use as a prognostic marker of significant coronary lesions remain to be elucidated.

Our findings emphasize the importance of the early onset of preventive efforts to control modifiable risk factors at a population scale with healthy lifestyle choices and medication when necessary. Although further research is required to confirm these findings, patients at a high risk estimated by cardiovascular risk functions may be candidates for noninvasive coronary angiography with computerized tomography scanning.

### Characteristics and Limitations of the Study

The cross-sectional nature of our design precluded the establishment of causal relationships. Although consecutively recruited, the population representativeness of our sample was limited for ethical reasons: only a small proportion of patients were asymptomatic, and the coronary angiography from which data were collected was only indicated for non-CAD cardiac surgery. The lipid profile, in particular cholesterol levels, was not measured; therefore, this important risk factor is missing from our analysis.

## 5. Conclusions

Our results suggest an association between some major cardiovascular risk factors and the presence of even relatively small asymptomatic coronary atherosclerotic lesions. The role of risk factors seems more determinant of such lesions in women. A novel finding is the independent inverse association of potassium levels with coronary atherosclerotic lesions in men but not in women, which deserves further attention and confirmation.

## Figures and Tables

**Table 1:** Demographic and clinical characteristics of participants by the presence of coronary atherosclerotic lesions with ≥30% stenosis (CALS-30).

	No CALS-30	≥1 CALS-30	*p*-Value
Characteristics	N = 632	N = 1010	
Age, years *	62.8 (11.9)	63.9 (11.6)	0.083
Men > 55 years/Women > 65 years	59.8%	68.3%	0.001
Sex: female	47.0%	25.6%	<0.001
Never-smoker/ex-smoker ≥ 1 year	88.2%	78.0%	<0.001
Ex-smoker < 1 year	4.9%	5.4%	
Current smoker	11.9%	22.0%	
Hypertension	55.9%	62.2%	0.013
BMI *	29.6 (12.6)	28.2 (4.94)	0.012
BMI > 30	38.7%	28.8%	<0.001
Previous PTCA	3.01%	5.74%	0.015
Diabetes or glycaemia > 126 mg/dL	34.2%	48.6%	<0.001
CKD or GFR < 60 mL/min	19.0%	23.2%	0.052
Laboratory determinations			
Creatinine mg/dL **	0.90 (0.71–1.05)	0.95 (0.80–1.15)	<0.001
BUN mg/dL **	16.4 (13.5–20.0)	16.4 (13.6–20.3)	0.702
GFR by CKD-EPI mL/min *	79.3 (22.6)	76.1 (24.0)	0.007
Potassium mmol/L **	4.40 (4.10–4.80)	4.30 (4.01–4.71)	0.009
Glycaemia mg/dL **	104 (94.0–122)	120 (100–154)	<0.001

BMI: body mass index. PTCA: percutaneous transcatheter coronary angioplasty. CKD: chronic kidney disease. BUN: blood urea nitrogen. GFR: estimated glomerular filtration rate. CKD-EPI: Chronic Kidney Disease Epidemiology Collaboration * Mean (standard deviation); ** Median [interquartile range].

**Table 2:** Conditions leading to coronary angiography by presence and severity of coronary atherosclerotic lesions with stenosis ≥ 30% (CALS-30).

	Non-CALS-30	≥1 CALS-30	*p*-Value
Reason for coronary angiography	N = 632	N = 1010	
ST-elevation myocardial infarction	9.34%	40.7%	<0.001
Non-ST-elevation myocardial infarction	11.6%	24.9%	
Unstable angina	11.7%	8.71%	
Chronic angina	24.2%	14.2%	
Preoperative valve surgery	13.9%	1.39%	
None of the above	29.3%	10.2%	

**Table 3:** The odds ratio of coronary lesions with ≥30% stenosis for classical cardiovascular risk factors. The effect is mutually adjusted and adjusted for age and sex in Model 1 and further adjusted for serum potassium in Model 2.

	Odds Ratio (95% Confidence Interval)	
	Model 1	Model 2
Age (one year)	1.02 (1.01; 1.03) *	1.02 (1.00; 1.03) *
Sex: Female	0.32 (0.25; 0.40) *	0.31 (0.25; 0.40) *
Ex-smoker <1 year	1.38 (0.86; 2.24)	1.32 (0.81; 2.16)
Current smoker	2.22 (1.63; 3.01) *	2.15 (1.56; 2.97) *
Hypertension	1.05 (0.83; 1.33)	1.00 (0.78; 1.28)
Diabetes	2.05 (1.63; 2.59) *	2.09 (1.64; 2.67) *
CKD or GFR < 60 mL/min	1.24 (0.94; 1.63)	1.42 (1.06; 1.90) *
Potassium		0.71 (0.58; 0.87) *

CKD: chronic kidney disease; GFR: estimated glomerular filtration rate; * *p*-value < 0.05.

**Table 4:** Association of a score based on the number of accumulated classical risk factors (diabetes, hypertension, chronic kidney disease, and current smoking) with patient characteristics and the presence of coronary atherosclerotic lesions with ≥30% stenosis (CALS-30).

# Risk Factors	0	1	2	3-4	*p*-Trend ***
	N = 407	N = 427	N = 536	N = 272	
CALS-30	51.9%	56.6%	66.2%	73.7%	<0.001
Age *	60.4 (12.9)	62.8 (11.9)	64.6 (10.2)	67.0 (11.2)	<0.001
Sex: female	5.8%	27.4%	39.4%	45.2%	<0.001
Body mass index *	27.7 (4.29)	29.0 (14.7)	29.2 (5.73)	29.2 (5.02)	0.016
Body mass index > 30	24.1%	32.8%	37.6%	34.9%	<0.001
Previous PTCA	2.95%	5.85%	4.10%	6.62%	0.103
Creatinine mg/dL **	0.88 [0.76; 0.99]	0.91 [0.80; 1.10]	0.90 [0.77; 1.07]	1.30 [1.00; 1.58]	<0.001
BUN mg/dL **	15.6 [13.0; 18.6]	16.1 [13.7; 19.1]	16.4 [13.6; 20.2]	19.6 [15.6; 23.8]	<0.001
GFR by CKD-EPI mL/min *	88.7 (15.3)	79.5 (20.9)	78.2 (20.8)	55.6 (27.8)	<0.001
Potassium mmol/L **	4.36 [4.10; 4.63]	4.34 [4.09; 4.70]	4.40 [4.09; 4.79]	4.40 [4.00; 5.00]	0.070
Glycaemia mg/dL **	99.5 [92.0; 108]	106 [94.0; 126]	130 [102; 171]	146 [116; 222]	<0.001

* Mean (standard deviation); ** Median [interquartile range]; *** Chi Square for trends (categorical variables) or linear regression (continuous variables). PTCA: percutaneous transcatheter coronary angioplasty; BUN: blood urea nitrogen; GFR: estimated glomerular filtration rate: CKD-EPI: Chronic Kidney Disease Epidemiology Collaboration.

**Table 5:** The odds ratio for a score based on the number of classical risk factors (hypertension, diabetes, chronic kidney disease, and smoking) of coronary atherosclerotic lesions with ≥30% stenosis, adjusted for age, sex (Model 1), and, additionally, potassium (Model 2).

	Odds Ratio (95% Confidence Interval)	
	Model 1	Model 2
Age (one year)	1.01 (1.00; 1.02) *	1.01 (1.00; 1.02) *
Sex: female	0.31 (0.25; 0.39) *	0.31 (0.24; 0.39) *
Cardiovascular risk score	1.50 (1.35; 1.67) *	1.51 (1.35; 1.69) *
Potassium		0.71 (0.58, 0.87) *

* *p*-value < 0.05. Model 1: N: 1642 (cases 1010); Area under the curve: 0.67 (0.64–0.70). Model 2: N: 1525 (cases 946); Area under the curve: 0.67 (0.65–0.70).

## Data Availability

Data is contained within the article and supplementary material.

## Supplementary Materials

The following are available online at https://www.mdpi.com/article/10.3390/jpm11080722/s1, Table S1: Characteristics of Subjects according to atherosclerotic coronary lesions with ≥30% stenosis CALS-30) in women, Table S2: Characteristics of Subjects according to atherosclerotic coronary lesions with ≥30% stenosis (CALS-30) in men, Table S3: Description of procedures on subjects by presence of coronary atherosclerotic lesions with >30% stenosis (CALS-30) in women, Table S4: Description of Procedures on subjects according to significant injuries in men, Table S5: Odds ratio of coronary atherosclerotic lesions with ≥ 30% stenosis for classical cardiovascular risk factors adjusted for age and potassium in women, Table S6: Odds ratio of coronary atherosclerotic lesions with ≥30% stenosis for classical cardiovascular risk factors adjusted for age and potassium in men, Table S7: Odds ratio of coronary atherosclerotic lesions with ≥ 30% stenosis for a cardiovascular risk score with the accumulation of diabetes, smoking, glomerular filtrate rate<60 min/ml and hypertension, adjusted for age and potassium in women, Table S8: Odds ratio of coronary atherosclerotic lesions with ≥ 30% stenosis for a cardiovascular risk score with the accumulation of diabetes, smoking, glomerular filtrate rate<60 ml/min and hypertension, adjusted for age and potassium in men, Table S9: Characteristics of Subjects according to coronary atherosclerotic lesions with ≥ 50% stenosis (CALS-50), Table S10: Characteristics of Subjects according to atherosclerotic coronary lesions with ≥70% stenosis (CALS-70), Table S11: Odds ratio of coronary atherosclerotic lesions with ≥ 50% stenosis for classical cardiovascular risk factors adjusted for age and sex (Model 1) and, additionally, potassium (Model 2), Table S12: Odds ratio of coronary atherosclerotic lesions with ≥ 70% stenosis for classical cardiovascular risk factors adjusted for age and sex (Model 1) and, additionally, potassium (Model 2), Table S13: Odds ratio of coronary atherosclerotic lesions with ≥ 50% stenosis for a cardiovascular risk score with the accumulation of diabetes, smoking, kidney disease or glomerular filtrate rate<60 ml/min and hypertension, adjusted for age and sex (Model 1) and, additionally, potassium (Model 2), Table S14: Odds ratio of coronary atherosclerotic lesions with ≥ 70% stenosis for a cardiovascular risk score with the accumulation of diabetes, smoking, kidney disease or glomerular filtrate rate<60 ml/min and hypertension, adjusted for age and sex (Model 1) and, additionally, potassium (Model 2).

## References

[1] Laslett L.J., Alagona P., Clark B.A., Drozda J.P., Saldivar F., Wilson S.R., Poe C., Hart M. (2012). The Worldwide Environment of Cardiovascular Disease: Prevalence, Diagnosis, Therapy, and Policy Issues. J. Am. Coll. Cardiol..

[2] Organization WH Cardiovascular Diseases. https://www.who.int/health-topics/cardiovascular-diseases/#tab=tab_1.

[3] Benjamin E.J., Virani S.S., Callaway C.W., Chamberlain A.M., Chang A.R., Cheng S., Chiuve S.E., Cushman M., Delling F.N., Deo R. (2018). Heart Disease and Stroke Statistics—2018 Update: A Report from the American Heart Association. Circulation.

[4] Khera A.V., Kathiresan S. (2017). Genetics of coronary artery disease: Discovery, biology and clinical translation. Nat. Rev. Genet..

[5] Mortensen M.B., Dzaye O., Steffensen F.H., Bøtker H.E., Jensen J.M., Sand N.P.R., Kragholm K.H., Sørensen H.T., Leipsic J., Mæng M. (2020). Impact of Plaque Burden Versus Stenosis on Ischemic Events in Patients with Coronary Atherosclerosis. J. Am. Coll. Cardiol..

[6] Solberg L.A., Strong J.P., Holme I., Helgeland A., Hjermann I., Leren P., Mogensen S.B. (1985). Stenoses in the coronary arteries. Relation to atherosclerotic lesions, coronary heart disease, and risk factors. The Oslo Study. Lab. Investig. J. Tech. Methods Pathol..

[7] Solberg L.A., Strong J.P. (1983). Risk factors and atherosclerotic lesions. A review of autopsy studies. Arter. Off. J. Am. Hear. Assoc. Inc..

[8] Berenson G.S., Wattigney W.A., Tracy R.E., Newman W.P., Srinivasan S.R., Webber L.S., Dalferes E.R., Strong J.P. (1992). Atherosclerosis of the aorta and coronary arteries and cardiovascular risk factors in persons aged 6 to 30 years and studied at necropsy (the Bogalusa Heart Study). Am. J. Cardiol..

[9] Mc Gill H.C., Mcmahan C.A., E Herderick E.E., Malcom G.T., E Tracy R.E., Strong J.P. (2000). for the Pathobiological Determinants of Atherosclerosis in Youth (PDAY) Research Group. Origin of atherosclerosis in childhood and adolescence. Am. J. Clin. Nutr..

[10] Bertoluci M.C., Rocha V.Z. (2017). Erratum to: Cardiovascular risk assessment in patients with diabetes. Diabetol. Metab. Syndr..

[11] Fuster V., Kelly B.B. (2010). Promoting Cardiovascular Health in the Developing World: A Critical Challenge to Achieve Global Health.

[12] McKibbin E.C. (1994). An analysis of the risk factors for coronary heart disease in patients aged 55 and younger with proven heart disease. Curationis.

[13] Icaza G., Núñez L., Marrugat J., Mujica V., Escobar M.C., Jiménez A.L., Pérez P., Palomo I. (2009). Estimation of coronary heart disease risk in Chilean subjects based on adapted Framingham equations. Rev. Med. Chile.

[14] Bambs C., Cerda J., Zarate V. (2007). Coronary heart disease in Chile. Lancet.

[15] Zhu X., Chen Y., Xiang L., You T., Jiao Y., Xu W., Chen J. (2018). The long-term prognostic significance of high-sensitive C-reactive protein to in-stent restenosis. Medicine.

[16] Levine G.N., Bates E.R., Blankenship J.C., Bailey S.R., Bittl J.A., Cercek B., Chambers C.E., Ellis S.G., Guyton R.A., Hollenberg S.M. (2016). 2015 ACC/AHA/SCAI Focused Update on Primary Percutaneous Coronary Intervention for Patients With ST-Elevation Myocardial Infarction: An Update of the 2011 ACCF/AHA/SCAI Guideline for Percutaneous Coronary Intervention and the 2013 ACCF/AHA Guideline for the Management of ST-Elevation Myocardial Infarction. J. Am. Coll. Cardiol..

[17] Thygesen K., Alpert J.S., Jaffe A.S., Simoons M.L., Chaitman B.R., White H.D. (2012). Third Universal Definition of Myocardial Infarction. Circulation.

[18] Levey A.S., Stevens L.A., Schmid C.H., Zhang Y.L., Castro A.F., Feldman H.I., Kusek J.W., Eggers P., Van Lente F., Greene T. (2009). A new equation to estimate glomerular filtration rate. Ann. Intern. Med..

[19] Botas J. (2003). Evaluación y guía terapéutica de las lesiones coronarias intermedias en el laboratorio de hemodinámica. Rev. Española Cardiol..

[20] Rosenthal R.L. (2015). The 50% Coronary Stenosis. Am. J. Cardiol..

[21] Jones E., Eteiba W., Merz N.B. (2012). Cardiac Syndrome X and Microvascular Coronary Dysfunction. Trends Cardiovasc. Med..

[22] Vaccarino V., Parsons L., Every N.R., Barron H.V., Krumholz H.M. (1999). Sex-Based Differences in Early Mortality after Myocardial Infarction. New Engl. J. Med..

[23] Chiha J., Mitchell P., Gopinath B., Plant A.J.H., Kovoor P., Thiagalingam A. (2015). Gender differences in the severity and extent of coronary artery disease. IJC Hear. Vasc..

[24] Legato M.J. (1997). Gender-specific physiology: How real is it? How important is it?. Int. J. Fertil. women’s Med..

[25] Appelman Y., van Rijn B.B., Haaf M.E.T., Boersma E., Peters S.A. (2015). Sex differences in cardiovascular risk factors and disease prevention. Atherosclerosis.

[26] Haffner S.M., Lehto S., Rönnemaa T., Pyörälä K., Laakso M. (1998). Mortality from Coronary Heart Disease in Subjects with Type 2 Diabetes and in Nondiabetic Subjects with and without Prior Myocardial Infarction. New Engl. J. Med..

[27] Manfrini O., Yoon J., van der Schaar M., Kedev S., Vavlukis M., Stankovic G., Scarpone M., Miličić D., Vasiljevic Z., Badimon L. (2020). Sex Differences in Modifiable Risk Factors and Severity of Coronary Artery Disease. J. Am. Hear. Assoc..

[28] Möller-Leimkühler A.M. (2007). Gender differences in cardiovascular disease and comorbid depression. Dialogues Clin. Neurosci..

[29] González-Clemente J.M., Palma S., Arroyo J., Vilardell C., Caixàs A., Giménez-Palop O., Delgado-Rodríguez M. (2007). Is diabetes mellitus a coronary heart disease equivalent? Results of a meta-analysis of prospective studies. Rev. Española Cardiol..

[30] Barua R.S., Ambrose J.A. (2013). Mechanisms of Coronary Thrombosis in Cigarette Smoke Exposure. Arter. Thromb. Vasc. Biol..

[31] Bagheri B., Radmard N., Faghani-Makrani A., Rasouli M. (2019). Serum Creatinine and Occurrence and Severity of Coronary Artery Disease. Med. Arch..

[32] Zalewska-Adamiec M., Bachorzewska-Gajewska H., Malyszko J., Malyszko J.S., Kralisz P., Tomaszuk-Kazberuk A., Hirnle T., Dobrzycki S. (2015). Chronic kidney disease in patients with significant left main coronary artery disease qualified for coronary artery bypass graft operation. Arch. Med. Sci..

[33] Kawabe M., Sato A., Hoshi T., Sakai S., Hiraya D., Watabe H., Kakefuda Y., Ishibashi M., Abe D., Takeyasu N. (2014). Impact of blood urea nitrogen for long-term risk stratification in patients with coronary artery disease undergoing percutaneous coronary intervention. IJC Hear. Vessel..

[34] Goyal A., Spertus J.A., Gosch K., Venkitachalam L., Jones P.G., Van den Berghe G., Kosiborod M. (2012). Serum Potassium Levels and Mortality in Acute Myocardial Infarction. JAMA.

[35] Hughes-Austin J.M., Rifkin D.E., Beben T., Katz R., Sarnak M.J., Deo R., Hoofnagle A.N., Homma S., Siscovick D.S., Sotoodehnia N. (2017). The Relation of Serum Potassium Concentration with Cardiovascular Events and Mortality in Community-Living Individuals. Clin. J. Am. Soc. Nephrol..

[36] Pun P.H., Goldstein B.A., Gallis J., Middleton J.P., Svetkey L.P. (2017). Serum Potassium Levels and Risk of Sudden Cardiac Death Among Patients With Chronic Kidney Disease and Significant Coronary Artery Disease. Kidney Int. Rep..

[37] Lai Y.-H., Leu H.-B., Yeh W.-T., Chang H.-Y., Pan W.-H. (2015). Low-normal serum potassium is associated with an increased risk of cardiovascular and all-cause death in community-based elderly. J. Formos. Med. Assoc..

[38] Sun Y., Byon C.H., Yang Y., Bradley W.E., Dell’Italia L.J., Sanders P.W., Agarwal A., Wu H., Chen Y. (2017). Dietary potassium regulates vascular calcification and arterial stiffness. JCI Insight.

[39] Cook N.R., Obarzanek E., Cutler J.A., Buring J.E., Rexrode K.M., Kumanyika S.K., Appel L.J., Whelton P.K., Trials of Hypertension Prevention Collaborative Research Group (2009). Joint Effects of Sodium and Potassium Intake on Subsequent Cardiovascular Disease: The Trials of Hypertension Prevention Follow-up Study. Arch. Intern. Med..

[40] Young D.B., Ma G. (1999). Vascular protective effects of potassium. Semin. Nephrol..

