# Supplementary material for: Association of Kidney Disease, Potassium, and Cardiovascular Risk Factor Prevalence with Coronary Arteriosclerotic Burden, by Sex
Source: J Pers Med. 2021 Jul 27;11(8):722. doi: 10.3390/jpm11080722 (PMC8400373; doi:10.3390/jpm11080722)
Supplement: Supplementary file 1 [file jpm-11-00722-s001.zip › jpm-1236482-supplementary.pdf]

Supplementary Information

Table S1: Characteristics of Subjects according to atherosclerotic coronary lesions with ≥30% stenosis CALS-30) in women.

Characteristics	No CALS-30 N=297	Any CALS-30 N= 259	p-value
**Age, years ***	64.9 (10.8)	67.3 (11.7)	**0.015**
**> 55 years Men/>65 Woman**	48.5%	56.4%	0.076
**Never-smoker / ex-smoker ≥ 1 year**	85.9%	78.4%	**0.010**
**Ex-smoker < 1 year**	4.04%	2.70%	
**Current smoker**	10.1%	18.9%	
**Hypertension**	61.6%	80.7%	**<0.001**
**BMI**	29.3 (5.84)	28.5 (5.51)	0.089
**BMI >30**	39.6%	32.9%	0.137
**Previous PTCA**	2.02%	6.56%	**0.014**
**Diabetes or glycemia > 126 mg/dl**	41.4%	64.9%	**<0.001**
**Kideney disease or eGFR<60 ml/min**	22.9%	35.5%	**0.001**
**Laboratory determinations**			
**Creatinine mg/dL ****	0.78 (0.67-0.92)	0.84 (0.70-1.11)	**0.001**
**BUN mg/dL ****	15.9 (12.6-19.6)	16.1 (13.2-20.2)	0.177
**Potassium mmol/L ****	4.40 (4.00-4.70)	4.30 (3.95-4.70)	0.240
**Glycemia mg/dL ****	106 (93.0-131)	133 (105-192)	**<0.001**
**GFR by CKD-EPI ml/min ***	77.5 (22.5)	68.7(26.2)	**<0.001**

GRF: estimated glomerular filtration rate**, CKD-EPI:** Chronic Kidney Disease Epidemiology Collaboration; **PTCA:** percutaneous transcatheter coronary angioplasty.

* Mean (standard deviation), ** Median (Interquartile range)

Table S2: Characteristics of Subjects according to atherosclerotic coronary lesions with ≥30% stenosis (CALS-30) in men.

Characteristics	No CALS-30 N=335	Any CALS-30 N= 259	p-value
**Age, years ***	61.0 (12.4)	62.7 (11.3)	**0.031**
**> 55 years Men/>65 Woman**	69.9%	72.4%	0.424
**Never-smoker / ex-smoker ≥ 1 year**	80.9%	70.6%	**<0.001**
**Ex-smoker < 1 year**	5.67	6.26%	
**Current smoker**	13.4%	23.2%	
**Hypertension**	50.7%	55.8%	0.140
**Body mass index ***	29.8 (16.3)	28.1 (4.73)	0.073
**Body mass index >30**	37.9%	27.4%	**0.001**
**Previous PTCA**	3.88%	5.46%	0.340
**Diabetes or glycemia > 126 mg/dl**	27.8%	43.0%	**<0.001**
**Kideney disease or eGFR<60 ml/min**	17.6%	35.5%	0.172
**Laboratory determinations**			
**Creatinine mg/dL ****	0.99 (0.81-1.12)	0.84 (0.70-1.11)	0.291
**BUN mg/dL ****	17.0 (14.4-20.1)	16.1 (13.2-20.2)	0.171
**Potassium mmol/L ****	4.46 (4.20-4.80)	4.30 (3.95-4.70)	**0.002**
**Glycemia mg/dL ****	103 (94.0-117)	133 (105-192)	**<0.001**
**GFR by CKD-EPI ml/min ***	81.0 (22.6)	68.7(26.2)	0.118

GRF: estimated glomerular filtration rate**, CKD-EPI:** Chronic Kidney Disease Epidemiology Collaboration; **PTCA:** percutaneous transcatheter coronary angioplasty.

* Mean (standard deviation), ** Median (Interquartile range)

Table S3: Description of procedures on subjects by presence of coronary atherosclerotic lesions with >30% stenosis (CALS-30) in women.

	**No CALS-30** **N=297**	**Any CALS-30** **N=259**	p-value
**Reason for study inclusion**			
**ST elevation myocardial infarction**	6.06%	33.6%	**<0.001**
**Non-ST-elevation myocardial infarction**	14.5%	24.7%	
**Unstable Angina**	12.5%	9.27%	
**Chronic Angina**	26.9%	18.5%	
**Preoperative valve surgery**	17.2%	1.93%	
**None of the above**	22.9%	12.0%	

Table S4: Description of Procedures on subjects according to significant injuries in men.

	No CALS-30<br>N=335	≥ 1 CALS-30<br>N=751	p-value
**Reason for study inclusion**			
**ST elevation myocardial infarction**	12.2%	43.1%	**<0.001**
**Non-ST-elevation myocardial infarction**	8.96%	24.9%	
**Unstable Angina**	11.0%	8.52%	
**Chronic Angina**	21.8%	12.6%	
**Preoperative valvular surgery**	11.0%	1.20%	
**None of the above**	34.9%	9.59%	

Table S5: Odds ratio of coronary atherosclerotic lesions with ≥ 30% stenosis for classical cardiovascular risk factors adjusted for age and potassium in women.

	**Odds ratio (95% confidence interval)**	
	Model 1	Model 2
**Age**	1.02 (1.00; 1.03)	1.02 (1.00; 1.03)
**Ex-smoker ≥ 1 year**	0.99 (0.37; 2.68)	0.77 (0.27; 2.19)
**Smoker / Ex-smoker < 1 year**	2.32 (1.37; 3.93)	2.11 (1.23; 3.63)
**Hypertension**	1.62 (1.05; 2.50)	1.56 (1.00; 2.44)
**Diabetes**	2.14 (1.47; 3.12)	2.09 (1.41; 3.10)
**Kidney disease or eGFR<60 ml/min**	1.46 (0.96; 2.21)	1.59 (1.03; 2.47)
**Potassium**		0.79 (0.57; 1.10)

Model 1: N: 556 (cases 259), **AUC:** 0.684 (0.639-0.728)
Model 2 further adjusted for potassium: **N:** 514 (cases 243), **AUC:** 0.679 (0.633-0.725)

Table S6: Odds ratio of coronary atherosclerotic lesions with ≥30% stenosis for classical cardiovascular risk factors adjusted for age and potassium in men.

	**Odds ratio (95% confidence interval)**	
	Model 1	Model 2
**Age**	1.02 (1.00; 1.03)	1.02 (1.00; 1.03)
**Ex-smoker ≥ 1 year**	1.58 (0.88; 2.84)	1.59 (0.88; 2.86)
**Smoker / Ex-smoker < 1 year**	2.20 (1.48; 3.27)	2.21 (1.49; 3.29)
**Hypertension**	0.82 (0.60; 1.10)	0.82 (0.60; 1.10)
**Diabetes**	2.05 (1.50; 2.80)	2.04 (1.50; 2.79)
**Kidney disease or eGFR<60 ml/min**	1.27 (0.86; 1.87)	1.34 (0.93; 1.94)
**Potassium**		0.67 (0.51; 0.87)

Model 1: N: 1086 (cases 751), **AUC**: 0.628 (0.592-0.664)
Model 2 further adjusted for potassium: **N**: 1011 (cases 703), **AUC**: 0.647 (0.610-0.683)

Table S7: Odds ratio of coronary atherosclerotic lesions with ≥ 30% stenosis for a cardiovascular risk score with the accumulation of diabetes, smoking, glomerular filtrate rate<60 min/ml and hypertension, adjusted for age and potassium in women.

	**Odds ratio (95% confidence interval)**	
	Model 1	Model 2
**Age**	1.01 (1.00; 1.03)	1.01 (1.00; 1.03)
**Cardiovascular Risk SCORE**	1.84 (1.53; 2.20)	1.82 (1.51; 2.20)
**Potassium**		0.79 (0.58; 1.10)

Model 1: N: 556 (cases 259), **AUC**: 0.678 (0.634-0.723)
Model 2 further adjusted for potassium: N: 514 (cases 243), AUC: 0.673 (0.627-0.720)

Table S8: Odds ratio of coronary atherosclerotic lesions with ≥ 30% stenosis for a cardiovascular risk score with the accumulation of diabetes, smoking, glomerular filtrate rate<60 ml/min and hypertension, adjusted for age and potassium in men.

	**Odds ratio (95% confidence interval)**	
	Model 1	Model 2
**Age**	1.01 (1.00; 1.02)	1.01 (1.00; 1.02)
**Cardiovascular Risk SCORE**	1.33 (1.17; 1.52)	1.36 (1.18; 1.56)
**Potassium**		0.66 (0.51; 0.86)

Model 1: N: 1086 (cases 751), **AUC**: 0.591 (0.554-0.627)
Model 2 further adjusted for potassium: N: 1011 (cases 703), **AUC:** 0.610 (0.572-0.648

Table S9: Characteristics of Subjects according to coronary atherosclerotic lesions with ≥ 50% stenosis (CALS-50).

Characteristics	No CALS-50<br>N= 673	≥ 1 CALS-50<br>N= 969	p-value
**Age, years ***	63.0 (12.1)	63.8 (11.5)	0.159
**> 55 years Men/>65 Woman**	60.3%	68.3%	**0.001**
**Sex: woman**	45.8%	25.6%	**<0.001**
**Non-smoker / Ex-smoker ≥ 1 year**	83.2%	72.1%	**<0.001**
**Ex-smoker < 1 year**	4.6%	5.6%	
**Current smoker**	12.2%	22.3%	
**Hypertension**	55.1%	63.0%	**0.002**
**Body mass index**	29.5 (12.2)	28.2 (4.97)	**0.016**
**Body mass index >30**	38.1%	28.8%	**<0.001**
**Previous PTCA**	3.27%	5.68%	**0.032**
**Diabetes or glycemia > 126 mg/dl**	34.8%	48.8%	**<0.001**
**Kidney disease or eGFR<60 ml/min**	19.9%	22.7%	0.196
**Laboratory determinations**			
**Creatinine mg/dL ****	0.90 (0.72;1.07)	0.95 (0.80;1.15)	**<0.001**
**BUN mg/dL ****	16.4 (13.5;20.0)	16.4 (13.6;20.2)	0.884
**Potassium mmol/L ****	4.40 (4.11;4.80)	4.30 (4.00;4.70)	**0.001**
**Glycemia mg/dL ****	104 (94.0;123)	121 (100;157)	**<0.001**
**GFR by CKD-EPI ml/min ***	78.8 (23.2)	76.3 (23.7)	**0.037**

GFR: estimated glomerular filtration rate**, CKD-EPI:** Chronic Kidney Disease Epidemiology Collaboration; **PTCA:** percutaneous transcatheter coronary angioplasty.

* Mean (standard deviation), ** Median (Interquartile range)

Table S10: Characteristics of Subjects according to atherosclerotic coronary lesions with ≥70% stenosis (CALS-70).

Characteristics	No CALS-70<br>N= 754	≥ 1 CALS-70<br>N= 888	p-value
**Age, years** *	63.3 (12.1)	63.6 (11.4)	0.601
**> 55 years Men/>65 Woman**	61.7%	67.9%	**0.010**
**Sex: woman**	44.4%	24.9%	**<0.001**
**Non-smoker / Ex-smoker ≥ 1 year**	83.3%	71.1%	**<0.001**
**Ex-smoker < 1 year**	4.2%	6.0	
**Smoker**	12.5%	23.0%	
**Hypertension**	34.2%	46.3%	**<0.001**
**Body mass index** *	29.4 (11.9)	28.2 (4.44)	**0.016**
**Body mass index >30**	36.6%	29.2%	**0.002**
**Previous PTCA**	4.24%	5.07%	0.503
**Diabetes or glycemia > 126 mg/dl**	36.2%	48.9%	**<0.001**
**Renal Failure or eGFR<60 ml/min**	20.8%	22.2%	0.543
**Laboratory determinations**			
**Creatinine mg/dL** **	0.90 [0.73;1.10]	0.95 [0.80;1.14]	**<0.001**
**BUN mg/dL** **	16.6 [13.5;20.0]	16.4 [13.6;20.0]	0.712
**Potassium mmol/L** **	4.40 [4.10;4.80]	4.30 [4.01;4.70]	**0.007**
**Glycemia mg/dL** **	105 [94.0;125]	121 [100;159]	**<0.001**
**GFR by CKD-EPI ml/min** *	78.2 (23.6)	76.6 (23.5)	0.198

GFR: estimated glomerular filtration rate**, CKD-EPI:** Chronic Kidney Disease Epidemiology Collaboration; **PTCA:** percutaneous transcatheter coronary angioplasty.

* Mean (standard deviation), ** Median (Interquartile range)

Table S11: Odds ratio of coronary atherosclerotic lesions with ≥ 50% stenosis for classical cardiovascular risk factors adjusted for age and sex (Model 1) and, additionally, potassium (Model 2).

	**Odds ratio (95% confidence interval)**	
	**Model 1**	**Model 2**
**Age**	1.02 (1.01; 1.02)	1.01 (1.00; 1.02)
**Sex: women**	0.34 (0.27; 0.43)	0.331 (0.26; 0.42)
**Ex-smoker ≥ 1 year**	1.56 (0.97; 2.52)	1.51 (0.93; 2.47)
**Smoker / Ex-smoker < 1 year**	2.12 (1.57; 2.85)	2.04 (1.50; 2.79)
**Hypertension**	1.17 (0.92; 1.48)	1.12 (0.88; 1.44)
**Diabetes**	1.95 (1.55; 2.45)	1.99 (1.57; 2.53)
**Kidney disease or GFR<60 ml/min**	1.13 (0.86; 1.47)	1.29 (0.97; 1.71)
**Potassium**		0.67 (0.55; 0.83)

Model 1: N: 1642 (cases 969), **AUC**: 0.673 (0.646-0.700)
Model 2 further adjusted for potassium: **N:** 1525 (cases 905), **AUC**: 0.68 (0.653-0.708)

Table S12: Odds ratio of coronary atherosclerotic lesions with ≥ 70% stenosis for classical cardiovascular risk factors adjusted for age and sex (Model 1) and, additionally, potassium (Model 2).

	Odds ratio (95% confidence interval)					
	Model 1			Model 2		
**Age**	1.01	1.00	1.02	1.01	1.00	1.02
**Sex: women**	0.36	0.29	0.45	0.36	0.28	0.45
**Ex-smoker ≥ 1 year**	1.78	1.11	2.85	1.71	1.05	2.77
**Smoker / Ex-smoker < 1 year**	2.15	1.61	2.87	2.05	1.52	2.77
**Hypertension**	1.08	0.86	1.36	1.03	0.81	1.31
**Diabetes**	1.88	1.50	2.35	1.94	1.54	2.45
**Kidney disease or GFR<60 ml/min**	1.07	0.82	1.39	1.18	0.90	1.56
**Potassium**				0.74	0.60	0.90

Model 1: N: 1642 (cases 888), **AUC**: 0.665 (0.639-0.692)
Model 2 further adjusted for **potassium**: **N:** 1525 (cases 830), **AUC**: 0.672 (0.645-0.699)

Table S13: Odds ratio of coronary atherosclerotic lesions with ≥ 50% stenosis for a cardiovascular risk score with the accumulation of diabetes, smoking, kidney disease or glomerular filtrate rate<60 ml/min and hypertension, adjusted for age and sex (Model 1) and, additionally, potassium (Model 2).

	**Odds ratio (95% confidence interval)**	
	**Model 1**	**Model 2**
**Age**	1.01 (1.00; 1.02)	1.01 (1.00; 1.02)
**Sex: women**	0.33 (0.27; 0.42)	0.33 (0.26; 0.41)
**CV Risk SCORE**	1.48 (1.34; 1.65)	1.50 (1.35; 1.68)
**Potassium**		0.67 (0.55; 0.81)

Model 1: N: 1642 (cases 969), **AUC**: 0.660 (0.633-0.687), **1-residual/null deviance**: 0.061, **CV** Cardiovascular;
Model 2 further adjusted for potassium: N: 1525 (cases 905), AUC: 0.667 (0.639-0.694), residual/null deviance: 0.067

Table S14: Odds ratio of coronary atherosclerotic lesions with ≥ 70% stenosis for a cardiovascular risk score with the accumulation of diabetes, smoking, kidney disease or glomerular filtrate rate<60 ml/min and hypertension, adjusted for age and sex (Model 1) and, additionally, potassium (Model 2).

	**Odds ratio (95% confidence interval)**	
	**Model 1**	**Model 2**
**Age**	1.00 (0.99; 1.01)	1.00 (0.99; 1.01)
**Sex: women**	0.35 (0.28; 0.44)	0.35 (0.28; 0.44)
**CV Risk SCORE**	1.42 (1.28; 1.57)	1.43 (1.28; 1.59)
**Potassium**		0.72 (0.60; 0.88)

Model 1: N: 1642 (cases 888), **AUC**: 0.647 (0.621-0.674), **1-residual/null deviance**: 0.053, **CV** Cardiovascular;

Model 2 further adjusted for potassium: N: 1525 (cases 830), AUC: 0.654 (0.626-0.681), residual/null deviance: 0.056
